# Combined lime and biochar application enhances cowpea growth and yield in tropical Alfisol

**DOI:** 10.1038/s41598-024-52102-7

**Published:** 2024-01-16

**Authors:** Aruna Olasekan Adekiya, Bolajoko Bisola Ayorinde, Timothy Ogunbode

**Affiliations:** 1 Agriculture Programme, College of Agriculture, Engineering and Science, Bowen University, Iwo, Osun State Nigeria; 2 College of Agricultural Sciences, Landmark University, PMB 1001, Omu-Aran, Kwara State Nigeria

**Keywords:** Plant sciences, Environmental sciences

## Abstract

It is essential to increase the pH of tropical soils in order to reduce acidity and promote soil and crop productivity. Therefore, experiments were carried out in 2020 and 2021 to assess the impacts of biochar and lime on the chemical properties, growth, nodulation, and yield of cowpea (*Vigna unguiculata*). The study involved various levels of lime (CaCO_3_) and wood biochar (ranging from 0 to 10 t ha^−1^), organized in a factorial combination. The treatments were arranged in a randomized complete block design and replicated three times. The application of lime and biochar, either separately or in combination, led to improvements in soil chemical properties such as pH, nitrogen (N), phosphorus (P), potassium (K), calcium (Ca), sodium (Na), magnesium (Mg), and cation exchange capacity (CEC), as well as enhancements in the growth, nodulation, and yield of cowpea when compared to the control. Lime and biochar alone and combined reduced exchangeable acidity (Al + H) relative to the control. Cowpea yield increased with lime rate up to a point, but then decreases. The highest cowpea yield is achieved at a lime rate of 2.5 t ha^−1^, whereas cowpea yield increased as the Biochar rate increased from 0 up to 10 t ha^−1^. There was a significant correlation between pH and cowpea pod weight in both years (2020 and 2021). The R values were − 0.615 and − 0.444 for years 2020 and year 2021 respectively at *P* < 0.05. At higher lime levels combined with biochar, there were considerable reductions in cowpea yield, and this decrease can be attributed to unfavorable soil pH conditions. Relative to 2.5 t ha^−1^ lime + 5 t ha^−1^ biochar, 10 t ha^−1^ lime + 10 t ha^−1^ biochar, reduced cowpea grain yield by 853% in 2020 and 845% in 2021. Since there were no significant differences between the effects of 2.5 t ha^−1^ lime + 5 t ha^−1^ biochar, 2.5 t ha^−1^ lime + 7.5 t ha^−1^ biochar, and 2.5 t ha^−1^ lime + 10 t ha^−1^ biochar applications on cowpea yield, therefore to prevent waste of Biochar, 2.5 t ha^−1^ lime + 5 t ha^−1^ biochar is recommended for production of cowpea.

## Introduction

Estimations indicate that a significant portion, over 25%, of the world's land is covered by acidic soils, and half of potentially fertile land is affected by this acidity issue^1^. This includes tropical soils as well^2^. In tropical regions, characterized by heavy rainfall and fast organic matter breakdown, nutrient loss, especially alkali cations are common^3^. Soil acidity has detrimental effects, reducing the availability of vital plant nutrients like phosphorus and molybdenum, while increasing the presence of toxic elements like aluminum and manganese to harmful levels^4^. The acidic conditions can harm the soil ecosystem, making it challenging for bacteria, earthworms, and other soil microbes to survive. Severe soil acidity can even inhibit helpful bacteria like rhizobia, crucial for legume nitrogen fixation^5^. Lower soil pH leads to decreased crop yields for many plants, cowpea being no exception^6^.

To boost soil and crop productivity, it's imperative to raise the soil's pH to levels suitable for optimal plant growth. Typically, soil acidity is addressed by applying agricultural lime, which raises the pH by countering the acidity. Liming involves using calcium- and magnesium-rich materials like marl, chalk, limestone, or hydrated oxide in various forms^7^. Although the application of agricultural lime is the primary method for mitigating soil acidity, in tropical countries like Nigeria, many farmers face challenges due to the associated costs^8^. Hence, finding an affordable soil amendment that can effectively raise soil pH, comparable to conventional agricultural lime, is essential.

Biochar, a carbon-rich substance produced by heating organic biomass with low oxygen levels, has emerged as a viable option^9^. Incorporating biochar into the soil acts as a liming substance, reducing soil acidity effectively^3^. This has been attributed to the liming potential of biochar due to high inherent pH of biochar, base cation content, CaCO_3_ content and calcium carbonate equivalent^10^. Moreover, biochar enhances soil aggregate stability, increases nutrient content and availability, and improves microbial activities in the soil, thereby enhancing soil fertility and aiding in the decomposition of organic matter^3^. Juriga and Šimanský^11^ showed that application of biochar at the rate of 20 t ha^−1^ significantly increased the values of soil pH (H_2_O) and soil pH (KCl) compared to control. Study was conducted to understand the effect of biochar (*Eucalyptus* wood, bamboo, and rice husk) on soil pH, soluble and exchangeable Al in soil with and without Al addition^12^. Wood biochar application to soil at 20 t ha^−1^ was found to be highly consistent in decreasing soil acidity and reducing soluble and exchangeable Al. Also, Lin et al.^13^ also reported an increased soil pH owing to the application peanut shell biochar to highly acidic red soil.

Cowpea (*Vigna unguiculata* (L.) Walp.) is a vital grain legume crop, particularly in the dry savannah regions of West Africa, contributing significantly to human nutrition, food security, and income generation for both farmers and food vendors in the region^14^. In Nigeria, cowpea constitutes about 40% of the daily protein consumption^15^. However, the performance of cowpea is greatly influenced by the pH of the soil^3^.

The optimum soil pH ranges from 5.5 to 6.5 is ideal for cowpea cultivation^16^. However, profile pits collected from Northern Guinea and Sudan savanna zones which are the major cowpea growing areas of northern Nigeria recorded a pH range of 4.6–6.8 (water) and 4.2–5.6 (CaCl_2_)^17^. In another study conducted to assess the spatial variability of pH and primary nutrients of Alfisols in north-eastern Nigeria^18^, the soil was acidic with a mean pH of 4.95 in water. These pointing to the fact that there is a gap between the ideal pH for cowpea cultivation and the soil pH status.

For the expansion and increased production of cowpea in Nigeria, while also reducing production costs, enhancing soil productivity is crucial. Consequently, raising the pH of tropical soils to mitigate acidity is necessary, ensuring both short-term and long-term soil productivity. Determining the optimal lime rate for cowpea production in the agro-ecological zone is essential to ensure profitable farming by avoiding lime wastage or reductions in crop yield^19^. Furthermore, it has been reported that combining organic and inorganic soil amendments increases crop yield better than their sole forms^20–22^. Studies on integrating biochar with lime have not been conducted. This integration is expected to reduce the lime rate and therefore, the cost. In a field experiment, husk biochar + limestone caused a significant increase of soil pH by about 28.2% relative to no amendment addition^23^. Other previous studies have shown that the combined use of biochar and inorganic fertilizers or amendments enhances yield compared to their singular applications^3,24^. Therefore, the objective of this study was to evaluate the effects of sole and combined applications of biochar and lime on soil chemical characteristics, growth, and yield of cowpea in tropical derived savanna Alfisol.

## Results

### Soil physical and chemical characteristics of the sites before sowing of cowpea and chemical characteristics of the biochar used for the study

Table 1 displays the physical and chemical properties of the soils at the experimental locations before the 2020 and 2021 experiments. In both years, the soils had a sandy loam texture. The sand content was 68.2% in 2020 and 68.1% in 2021. Silt content remained constant at 16.1% for both years. Clay content was 15.7% in 2020 and 15.8% in 2021. The pH of the soils in water was 5.33 (2020) and 5.30 (2021), both indicating strong acidity^25^. Exchangeable calcium was 0.99 cmol kg^−1^ for both years. Magnesium content was 0.81 cmol kg^−1^ in 2020 and 0.82 cmol kg^−1^ in 2021, indicating a high concentration. Exchangeable potassium was low at 0.14 cmol kg^−1^ for both years. Exchangeable sodium was 0.01 cmol kg^−1^ at both sites. Organic carbon was 1.13% in 2020 and 1.14% in 2021, indicating a generally low organic matter content. Phosphorus levels were low (9.2 mg kg^−1^ in 2020 and 9.6 mg kg^−1^ in 2021), as were nitrogen levels (0.11% in 2020 and 0.15% in 2021), falling below the critical levels recommended for crop production in Nigeria's ecological zones^26^. The cation exchange capacity (CEC) was 9.2 cmol kg^−1^ in 2020 and 9.9 cmol kg^−1^ in 2021.

**Table 1:** Soil physical and chemical properties of the experimental sites prior to planting.

Property	2020	2021
Sand (%)	68.2	68.1
Silt (%)	16.1	16.1
Clay (%)	15.7	15.8
Textural class	Sandy loam	Sandy loam
Organic C (%)	1.13	1.14
pH (water)	5.33	5.30
pH (KCL)	5.28	5.28
N (%)	0.11	0.15
P (mg kg^−1^)	9.2	9.6
K (cmol kg^−1^)	0.14	0.14
Ca(cmol kg^−1^)	0.99	0.99
Mg (cmol kg^−1^)	0.81	0.82
Na (cmol kg^−1^)	0.11	0.11
(Al + H) (cmol kg^−1^)	1.80	1.82
ECEC (cmol kg^−1^)	3.85	3.88

Table 2 illustrates the pH and composition of the biochar. The pH was 7.31, indicating a slightly alkaline nature^25^ that could potentially help raise the soil pH. The biochar had an ash content of 0.5% and a relatively high organic carbon content of 61.5%, suggesting it could enhance the soil's carbon content. Additionally, it contained nitrogen (0.81%), phosphorus (0.69%), potassium (1.39%), calcium (1.20%), magnesium (0.40%), and sodium (0.41%). The carbon-to-nitrogen (C:N) ratio of the biochar was 75.92.

**Table 2:** Chemical characteristics of the biochar used for the experiment.

Property	Value
pH (water)	7.31
Ash (%)	0.50
Organic C (%)	61.5
N (%)	0.81
P (%)	0.69
K (%)	1.39
Ca (%)	1.20
Mg (%)	0.40
Na (%)	0.41
C:N	75.92

### Effect of lime and biochar on soil chemical properties

The impacts of lime and biochar on soil chemical properties are detailed in Tables 3 and 4. Analyzing lime and biochar separately for both years (2020 and 2021), lime application had a significant effect on soil pH and available phosphorus (P). Both pH and P levels increased with higher lime application rates (from 0 to 10 t ha^−1^). Lime also notably influenced soil nitrogen (N) content at a 7.5 t ha^−1^ application. However, soil organic carbon (OC) was not significantly affected by lime application. Biochar, when applied alone, significantly impacted soil pH, OC, P, and N content as the level increased from 0 to 10 t ha^−1^. The combined effects of lime and biochar (L × B) were significant for pH, N, OC, and P as the application levels of both lime and biochar increased (Table 3).

**Table 3:** Effect of lime and biochar on soil chemical properties (pH, N, OC and P).

Lime (t ha^−1^)	Biochar (t ha^−1^)	pH(H_2_0)		N (%)		OC (%)		P (mg kg^−1^)	
		2020	2021	2020	2021	2020	2021	2020	2021
0.0	0.0	5.30r	5.28s	0.10i	0.14ef	1.11j	1.12j	9..00s	9.30s
0.0	2.5	5.30p	5.40r	0.14fg	0.16cd	1.14h	1.23cd	9.50r	9.80r
0.0	5.0	5.90n	5.90p	0.17bc	0.17bc	1.19de	1.24bc	10.30qr	10.60q
0.0	7.5	6.10m	6.20n	0.15df	0.18bc	1.23bc	1.26ab	10.80pq	11.40q
0.0	10.0	6.20lm	6.30l	0.16cd	0.19a	1.24b	1.28a	11.40op	12.40o
2.5	0.0	5.50o	5.80q	0.10i	0.13fg	1.11j	1.12jk	10.50pq	10.80q
2.5	2.5	5.60o	6.30l	0.10i	0.13fg	1.12ij	1.13ij	11.70o	11.70pq
2.5	5.0	6.10m	6.30l	0.14fg	0.14ef	1.13hi	1.15h	14.20n	13.40n
2.5	7.5	6.20lm	6.50k	0.17bc	0.16cd	1.24b	1.19e	16.80lm	15.80m
2.5	10.0	6.30kl	6.60i	0.19a	0.19a	1.28a	1.28a	18.50kl	17.70l
5.0	0.0	6.30kl	6.30 l	0.11hi	0.11i	1.12ij	1.12jk	15.90mn	18.80k
5.0	2.5	6.30kl	5.70q	0.12ji	0.12gh	1.13hi	1.13ij	17.40l	19.70j
5.0	5.0	6.40ij	6.10o	0.13hi	0.13fg	1.14h	1.14hi	19.60k	21.80i
5.0	7.5	6.50i	6.20n	0.15df	0.15de	1.19de	1.19e	20.80jk	23.60h
5.0	10	6.70h	6.30l	0.17bc	0.18ab	1.24b	1.25bc	22.30l	25.80g
7.5	0.0	6.50i	6.60i	0.18ab	0.17bc	1.13hi	1.12jk	23.40h	23.90h
7.5	2.5	6.60i	6.60j	0.17bc	0.18ab	1.17fg	1.17fg	24.60g	25.30g
7.5	5.0	6.80h	6.80h	0.13gh	0.19a	1.18ef	1.24bc	26.90f.	27.10f.
7.5	7.5	6.90g	6.80g	0.15df	0.19a	1.19de	1.25bc	28.20e	28.70ef
7.5	10.0	7.10f	7.00f	0.16fg	0.19a	1.20d	1.28a	30.20d	30.40d
10.0	0.0	7.20e	7.10e	0.14fg	0.16cd	1.13hi	1.23cd	28.10e	28.80e
10.0	2.5	7.40d	7.30d	0.15df	0.16cd	1.17fg	1.24bc	29.60de	30.10d
10.0	5.0	7.80c	7.70c	0.17ab	0.18ab	1.19de	1.26ab	32.40c	33.30c
10.0	7.5	8.40b	8.20b	0.18df	0.19de	1.24b	1.27ab	34.60b	35.80b
10.0	10.0	8.90a	8.30a	0.18a	0.19a	1.28a	1.28a	38.70a	37.50a
SD		1.58	0.76	0.03	0.03	0.05	0.06	9.40	8.78
*p* values									
Lime (L)		< 0.001	< 0.001	< 0.001	< 0.001	< 0.001	< 0.001	< 0.001	< 0.001
Biochar (B)		< 0.001	< 0.001	< 0.001	< 0.001	< 0.001	< 0.001	< 0.001	< 0.001
L × B		< 0.001	< 0.001	< 0.001	< 0.001	< 0.001	< 0.001	< 0.001	< 0.001

Values followed by similar letters under the same column are not significantly different at *p* = 0.05 according to Duncan’s multiple range test.

**Table 4:** Effect of lime and biochar on soil chemical properties (Na, Ca, Mg, K, Al + H and CEC).

Lime (t ha^−1^)	Biochar (t ha^−1^)	Na (cmol kg^−1^)		Ca (cmol kg^−1^)		Mg (cmol kg^−1^)		K (cmol kg^−1^)		Al + H (cmol kg^−1^)		ECEC (cmol kg^−1^)	
		2020	2021	2020	2021	2020	2021	2020	2021	2020	2021	2020	2021
0.0	0.0	0.10g	0.10p	0.98s	0.96v	0.78v	0.79s	0.10m	0.13l	1.90a	1.91a	3.86n	3.62l
0.0	2.5	0.10g	0.10p	1.28r	1.26u	1.38u	1.48r	0.42l	0.43k	1.80ab	1.80ab	4.98m	5.07k
0.0	5.0	0.20f	0.14o	1.67q	1.69t	1.57t	1.58q	0.44k	0.44jk	1.60cd	1.70bc	5.48l	5.55ij
0.0	7.5	0.21f	0.17n	1.78p	1.78s	1.88s	1.89p	0.43k	0.44jk	1.60	1.61cd	5.90k	5.89i
0.0	10.0	0.22ef	0.21m	2.23o	2.25r	2.23r	2.36o	0.45k	0.46j	1.50de	1.50d	6.63j	6.78
2.5	0.0	0.26e	0.25l	2.89n	2.99q	2.59q	2.60n	0.52hi	0.49i	1.80ab	1.81ab	8.06hi	8.14g
2.5	2.5	0.27ef	0.27k	2.99m	3.03p	2.79p	2.89m	0.54h	0.56fg	1.40ef	1.61cd	7.99hi	8.36g
2.5	5.0	0.27ef	0.28i	3.03l	3.05n	3.33k	3.33i	0.54h	0.58ef	1.40ef	1.40e	8.85fg	8.64f
2.5	7.5	0.27ef	0.28i	3.34i	3.37k	3.54i	3.54g	0.54h	0.59de	1.30fg	1.30ef	8.99fg	9.08e
2.5	10.0	0.28f	0.29i	3.65g	3.75h	3.75g	3.77e	0.55h	0.59de	1.20gh	1.20fg	9.43de	9.60c
5.0	0.0	0.32d	0.33h	3.01lm	3.04o	3.03o	3.05l	0.55h	0.53h	1.20hi	1.19g	8.11hi	8.14g
5.0	2.5	0.31d	0.33h	3.11k	3.13m	3.13n	3.23k	0.57g	0.54h	1.10ij	1.10h	8.22gh	8.33g
5.0	5.0	0.32d	0.35g	3.21j	3.24l	3.31l	3.33i	0.59e	0.56ef	1.10ij	1.07hi	8.53gh	8.59fg
5.0	7.5	0.33d	0.35g	3.33i	3.36k	3.33k	3.36i	0.61d	0.58ef	0.90jk	1.01j	8.50gh	8.66f
5.0	10	0.34d	0.36g	3.43h	3.44j	3.45j	3.46h	0.62cd	0.59de	0.80k	0.94k	8.64gh	8.79f
7.5	0.0	0.43c	0.42de	3.65g	3.67i	3.75g	3.77d	0.56fg	0.55gh	0.80k	0.81l	9.19ef	9.22de
7.5	2.5	0.40c	0.43d	3.78f	3.78g	3.76g	3.78d	0.57fg	0.58ef	0.7kl	0.75m	9.11ef	9.32de
7.5	5.0	0.41c	0.43d	3.87f	3.88f	3.88f	3.88d	0.61d	0.63bc	0.6lm	0.60n	9.47de	9.42de
7.5	7.5	0.41c	0.43de	3.87e	4.04e	4.12e	4.15c	0.62cd	0.64b	0.50m	0.31o	9.52de	9.57cd
7.5	10.0	0.41c	0.43de	4.05d	4.09d	4.15d	4.17c	0.62cd	0.67a	0.40n	0.21p	9.27ef	9.56cd
10.0	0.0	0.53b	0.54c	3.85e	3.88f	3.85f	3.88d	0.59e	0.61cd	0.00o	0.00q	8.84fg	8.91d
10.0	2.5	0.54b	0.56c	3.98f	3.99e	3.88f	3.89d	0.63bc	0.63bc	0.00o	0.00q	9.03f	9.07e
10.0	5.0	0.58a	0.58b	4.45c	4.51c	4.35c	4.65b	0.64bc	0.64b	0.00o	0.00q	10.02c	10.38b
10.0	7.5	0.58a	0.59ab	4.65b	4.65b	4.45b	4.65b	0.64ab	0.64b	0.00o	0.00q	10.32bc	10.53b
10.0	10.0	0.60a	0.60a	5.53a	5.52a	5.51a	5.55a	0.65a	0.65ab	0.00o	0.00q	12.29a	12.32a
SD		0.14	0.15	1.05	1.06	1.06	1.08	0.11	0.11	0.62	0.65	1.81	1.86
*p* values													
Lime (L)		< 0.001	< 0.001	< 0.001	< 0.001	< 0.001	< 0.001	< 0.001	< 0.001	< 0.001	< 0.001	< 0.001	< 0.001
Biochar (B)		< 0.001	< 0.001	< 0.001	< 0.001	< 0.001	< 0.001	< 0.001	< 0.001	< 0.001	< 0.001	< 0.001	< 0.001
L × B		< 0.001	< 0.001	< 0.001	< 0.001	< 0.001	< 0.001	< 0.001	< 0.001	< 0.001	< 0.001	< 0.001	< 0.001

Values followed by similar letters under the same column are not significantly different at *p* = 0.05 according to Duncan’s multiple range test.

Additionally, lime application significantly influenced sodium (Na), calcium (Ca), magnesium (Mg), potassium (K), and cation exchange capacity (CEC) in the soil, with increments observed from 0 to 10 t ha^−1^. Concurrently, it reduced soil acidity (Al + H) with increasing lime levels. Similarly, biochar, when applied alone, increased Na, Ca, Mg, CEC, and K content in the soil as the application level rose from 0 to 10 t ha^−1^. Biochar significantly reduced soil acidity when the application level increased. The combined effects of lime and biochar (L × B) were significant for Na, Ca, Mg, CEC, K, and acidity (Al + H) (Table 4).

### Effect of lime and biochar on the growth and yield parameters of cowpea

The effects of lime and biochar on the growth parameters (vine length per plant, number of leaves per plant, number of branches per plant, and leaf area per plant) are shown in Table 5. Taking lime and biochar as a single factor in both years (2020 and 2021). Both lime and biochar significantly influenced the vine length per plant, number of leaves per plant, and number of branches per plant relative to the control. The interactive effects of L × B were significant for vine length per plant, number of leaves per plant, and number of branches per plant when increased from 0 to10 t ha^−1^.

**Table 5:** Effect of lime and biochar on the growth parameters of cowpea.

Lime (t ha^−1^)	Biochar (t ha^−1^)	Shoot length (cm)		Number of leaves/plant		Branch length (cm)	
		2020	2021	2020	2021	2020	2021
0.0	0.0	105.9n	122.9i	420.0o	463.0n	140.0o	154.3m
0.0	2.5	163.7m	190.0h	662.0n	674.0m	220.7n	224.7l
0.0	5.0	174.7lm	203.7h	701.0m	740.0l	233.7m	246.7k
0.0	7.5	195.3kl	216.8hi	828.0l	843.0k	276.0l	281.0j
0.0	10.0	203.8jk	216.9hi	833.0l	848.0k	277.7l	282.7j
2.5	0.0	223.5ij	233.5gh	942.0k	1010.0j	314.0k	336.7i
2.5	2.5	225.2ij	233.2gh	981.0j	1015.0j	327.0j	338.3i
2.5	5.0	227.0hij	247.2fg	1022.0i	1043.0i	340.7i	347.0h
2.5	7.5	228.8fghi	248.6fg	1033.0i	1046.0i	344.3i	348.7h
2.5	10.0	235.2fgh	252.2fg	1176.0h	1250.0gh	392.0h	416.7g
5.0	0.0	235.2fgh	257.0efg	1206.0g	1256.0gh	402.0g	418.7fg
5.0	2.5	243.6gh	257.3efg	1225.0eg	1265.0gh	408.3fg	421.7fg
5.0	5.0	245.6fgh	258.5efg	1230.0ef	1266.0gh	410.0ef	422.0fg
5.0	7.5	246.8fgh	262.2efg	1236.0ef	1280.0fg	412.0ef	426.7ef
5.0	10.0	252.2fgh	262.3efg	1238.0ef	1293.0ef	412.6ef	431.0de
7.5	0.0	252.2fgh	263.5efg	1246.0ef	1300.0ef	415.3ef	433.3cd
7.5	2.5	257.2efg	267.2def	1252.0e	1309.0e	417.3e	438.0bc
7.5	5.0	258.8def	268.7def	1284.0d	1314.0e	428.0d	439.7bc
7.5	7.5	258.8def	275.4def	1305.0c	1319.0e	435.0c	446.7bc
7.5	10.0	268.8cde	286.9cde	1308.0c	1330.0cd	436.0c	449.3b
10.0	0.0	270.2cde	288.7bcd	1368.0b	1348.0c	456.0b	459.0a
10.0	2.5	276.8bcd	288.8bcd	1372.0b	1374.0b	457.3b	461.0a
10.0	5.0	278.7bc	290.1abc	1388.0ab	1381.0ab	462.7ab	464.3a
10.0	7.5	303.6ab	310.7ab	1394.0a	1387.0ab	464.7a	466.3a
10.0	10.0	306.8a	316.8a	1402.0a	1403.0a	467.3a	467.7a
SD		44.04	41.10	262.46	257.87	87.47	87.17
*p* values							
Lime (L)		0.00	0.00	0.00	0.00	0.00	0.00
Biochar (B)		0.00	0.00	0.00	0.00	0.00	0.00
L × B		0.00	0.00	0.00	0.00	0.00	0.00

Values followed by similar letters under the same column are not significantly different at *p* = 0.05 according to Duncan’s multiple range test.

**Figure 1:**
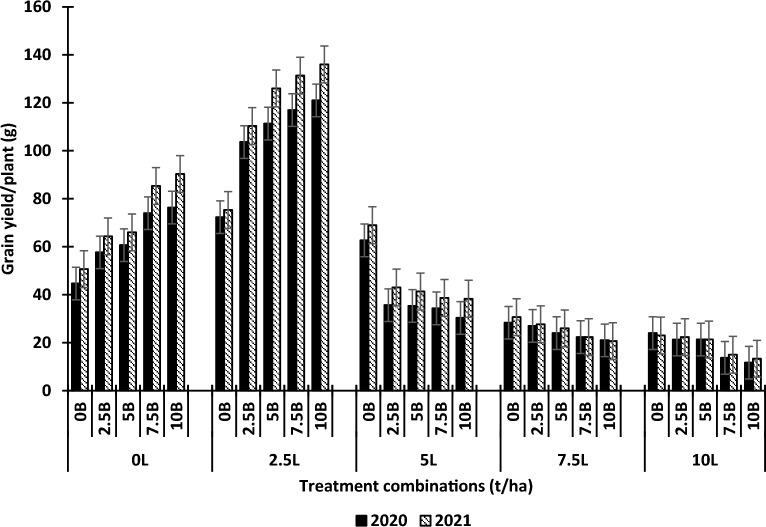
Effects of lime and biochar on grain yield of cowpea. Vertical bars show standard error of paired comparisons. *B* biochar; *L* lime.

**Figure 2:**
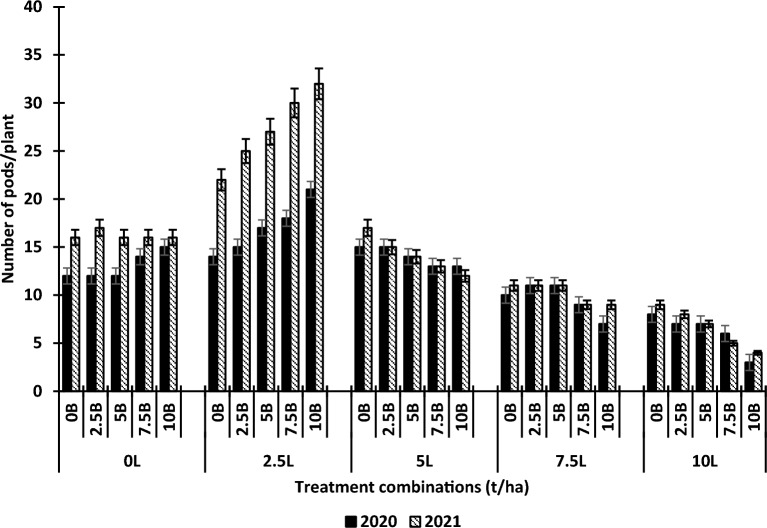
Effects of lime and biochar on number of pods of cowpea. Vertical bars show standard error of paired comparisons. *B* biochar, *L* lime.

The effects of lime and biochar on the grain yield per plant and number of pods per plant are shown in Figs. 1 and 2 respectively. Using lime and biochar as a single factor, Lime significantly influenced the grain yield of the plant relative to the control. There was a significant increase in the grain yield and the number of pods of the cowpea when lime was applied relative to the control. However, the increase in cowpea grain yield was only up to 2.5 t ha^−1^ while that of the number of pods was 5.0 t ha^−1^ lime after which there was a reduction in cowpea yield parameters. Biochar alone also significantly influenced the grain yield and the number of pods of cowpea relative to the control. The increase in these yield parameters with biochar was from 0 to 10 t ha^−1^. At higher lime levels combined with biochar, there were considerable reductions in cowpea yield, relative to 2.5 t ha^−1^ lime + 5 t ha^−1^ biochar, 10 t ha^−1^ lime + 10 t ha^−1^ biochar, reduced cowpea grain yield by 853% in 2020 and 845% in 2021. The differences in the effects of 2.5 t ha^−1^ lime + 5 t ha^−1^ biochar, 2.5 t ha^−1^ lime + 7.5 t ha^−1^ biochar, and 2.5 t ha^−1^ lime + 10 t ha^−1^ biochar on cowpea yield were not significant. The interactive effects of L × B were significant for grain yield and number of pods. In 2020, relative to the control (0 t ha^−1^ lime + 0 t ha^−1^ biochar), 2.5 t ha^−1^ lime + 0 t ha^−1^ biochar, 2.5 t ha^−1^ lime + 2.5 t ha^−1^ biochar, 2.5 t ha^−1^ lime + 5 t ha^−1^ biochar, 2.5 t ha^−1^ lime + 7.5 t ha^−1^ biochar, 2.5 t ha^−1^ lime + 10 t ha^−1^ biochar increased the grain yield of cowpea by 170.8%, 68%, 17.4%, 8.7%, and 3.4%, respectively. Also, in 2021, relative to the control (0 t ha^−1^ lime + 0 t ha^−1^ biochar), 2.5 t ha^−1^ lime + 0 t ha^−1^ biochar, 2.5 t ha^−1^ lime + 2.5 t ha^−1^ biochar, 2.5 t ha^−1^ lime + 5 t ha^−1^ biochar, 2.5 t ha^−1^ lime + 7.5 t ha^−1^ biochar, 2.5 t ha^−1^ lime + 10 t ha^−1^ biochar increased the grain yield of cowpea by 172.8%, 81%, 26%, 7.9%, and 3.8%, respectively.

### Effect of lime and biochar on the number of root nodules of cowpea

**Figure 3:**
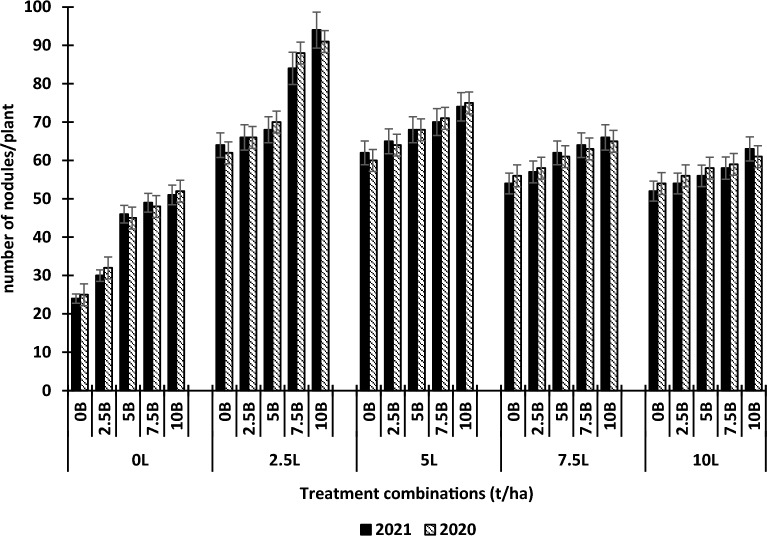
Effects of lime and biochar on number of nodules of cowpea. Vertical bars show standard error of paired comparisons. *B* biochar, *L* lime.

Figure 3 shows the effects of various combinations of different levels of lime and biochar on the number of nodules of cowpea. Lime and biochar individually increase the number of nodules of cowpea relative to the control. However, lime increased root nodules in this experiment up to 2.5 t ha^−1^ after which there was a decrease whereas biochar increased it up to 10 t ha^−1^. The highest number of root nodules of cowpea was attained at 2.5 t ha^−1^ lime + 10.0 t ha^−1^ biochar level.

## Discussion

The study revealed that the experimental sites exhibited low levels of OC, N, Ca, Na, and K, while Mg and P content were moderate. The soil pH at both sites was strongly acidic, a characteristic of soils found in the Nigerian savanna (see Table 1). This aligns with Adegbite et al.'s^27^ findings that Nigerian savanna soils have low organic matter and chemical fertility. The initial low cation exchange capacity (CEC) was attributed to the increased acidity (Al + H) of the sites before the experiment began.

Significant improvements in soil chemical properties were observed with the application of lime and biochar, either separately or in combination, compared to the control. Lime application raised the soil pH by displacing acidic ions (H^+^, Fe^2+^, Al^3+^, Mn^4+^, and Cu^2+^) from soil adsorption sites with Ca^2+^ ions from the lime (CaCO3), leading to an increase in pH^28^. The higher pH facilitated an increase in soil N content, as acidic conditions tend to fix essential nutrients like N, P, and K, making them less available to plants^4^. The increase in soil pH could lead to an increase in the amount of nitrogen in the soil because the rise in soil pH can lead to an increase in soil microorganisms associated with nitrogen fixation. Cowpea is characterized by its ability to host rhizobia bacteria, which contributes to nitrogen fixation for legume crops. On the other hand, acidic soils (the control) can make it difficult for beneficial bacteria to thrive and fix atmospheric nitrogen into the soil, resulting in less nitrogen in the soil. Lime application also enhanced the release of phosphate ions fixed by Al and Fe ions into the soil solution^29^, consequently increasing soil P levels.

Application of biochar alone significantly elevated soil pH, N, P, K, Ca, Mg, and OC content. Biochar's liming ability, with a pH of 7.31, and enrichment with basic cations, especially Ca, contributed to the higher pH in biochar-applied soil compared to the control. The increased organic C content in biochar-applied soil was attributed to the high carbon content in the wood feedstock. Biochar treatments improved soil N levels by retaining soil N effectively, reducing losses through leaching, and adsorbing cations and anions. The presence of carboxylate groups in biochar contributed to its high retention capacity^30^. Previous studies also support the significant increase in N with biochar application using different feedstocks^31,32^. Biochar application reduced P leaching, resulting in significantly higher P content in the soil compared to the control, thereby maintaining a reasonable level of P in the soil^33^.

The enhanced K, Ca, Mg and CEC of biochar applied soils could be due to the fact that the pyrolyzed wood feedstock used for this experiment contains a large amount of K, Ca, and Mg, hence high K, Ca and Mg in their soils. The noticeable increase in K, Ca, and Mg in soils treated with biochar can also be attributed to the presence of cation exchange sites on the biochar's surface. According to research by Jia et al.^34^, biochar has the ability to absorb leachate, aiding in the absorption of organic matter, total soluble N, plant-available P, and K. This contributes to an enhancement of the soil's nutrient retention capacity. Biochar particles possess colloidal properties, offering large specific surface areas and negative surface charges due to deprotonated functional groups. Consequently, nutrients dissolved in the soil solution are attracted to these colloidal surfaces. Studies by Jones et al.^35^ and Wang et al.^36^ have shown that biochar can increase total C, total N, and P content, as well as pH in the soil.

The cation exchange capacity (CEC) of the soil significantly increased with the sole application of biochar. This increase can be attributed to the high surface area and porous nature of biochar, as observed in the research by Nigussie et al.^31^. This provides an opportunity for Al and Fe to bind to the soil's exchange sites. Additionally, the reduction in exchangeable acidity, as noted by Agusalim et al.^37^, contributes to an increase in the soil's CEC content.

The combination of lime and biochar (L × B) can synergistically impact soil properties, including CEC. The alkaline properties of lime complement the CEC-enhancing properties of biochar, resulting in a greater overall increase in CEC compared to using them separately. Lime can also enhance the availability of certain nutrients, promoting their adsorption onto biochar and subsequently improving CEC.

The improved soil chemical properties observed with an increased biochar application rate are a result of the cumulative effects of biochar at each application level. This includes increased OC, N, P, K, Ca, Mg, Na, and CEC of the soils with varying biochar rates.

The sole application of biochar significantly improved nodulation at various biochar levels compared to the control. Biochar application helps improve the pH of acidic soils^38^, creating a more suitable environment for rhizobial activity and nodulation. Cowpeas, like many leguminous plants, have a symbiotic relationship with nitrogen-fixing bacteria called rhizobia, and maintaining an optimal soil pH is crucial for effective nitrogen fixation, growth, and nodulation. Furthermore, improved soil pH enhances the availability of soil phosphorus (P) for crop uptake, thus promoting better nodulation. Phosphorus is a critical nutrient for cowpea yield, stimulating growth, initiating nodule formation, and influencing the efficiency of rhizobium-legume symbiosis^39^.

The increase in cowpea growth and yield due to lime and biochar alone resulted from the improvement in soil chemical properties exhibited by lime and biochar. In this experiment, biochar and lime have raised soil pH and reduced acidity, making fixed phosphorus available for cowpea uptake. The addition of lime and biochar causes an elevation in the soil pH, facilitating an increase in the rate at which phosphate ions are released into the soil solution^29^. Thus, the application of lime in the form of biochar and CaCO_3_ aids in hydrolyzing aluminum and iron ions precipitated by phosphorus. Hence, the precipitated phosphate ion is released into the soil solution, making it available for plant absorption. Phosphorus is critical to cowpea yield because it stimulates growth, initiates nodule formation, and influences the efficiency of rhizobium-legume symbiosis^39^. Biochar has been reported to raise soil pH^40^, making fixed soil phosphorus more readily available for crop uptake and, consequently, better nodulation.

There was a significant correlation between pH and cowpea grain yield in both years. The R values were − 0.615 and − 0.444 for years 2020 and year 2021 respectively at *P* < 0.05. This implies that the yield of cowpea in this experiment is dependent on soil pH. Lime at 2.5 t ha^−1^ with any level of biochar is adequate for cowpea in this experiment. The increase in the pH of the soil at varying levels explains why there was an increase in the yield of the crop at the mentioned levels. Cowpea is an important legume crop that will only do well in a pH that is between 5.5 and 6.5^16^. Soil characteristics, such as pH and Al^3+^ may compromise symbiotic efficiency and plant development. pH values below 5.0 are reported to be deleterious for nodulation and nitrogen fixation hence, it will restrict the growth of cowpea^41^. Therefore, the reason for the increase in the yield parameter can be traced to having a favorable soil pH condition.

The interaction between lime and biochar also influenced the yield parameters of cowpea. At higher levels of lime (10 t ha^−1^) with biochar, there were drastic yield reductions of cowpea, the reduction in the yield of cowpea can best be explained by the presence of unfavorable soil pH conditions. For example, at 10 t ha^−1^ biochar and 10 t ha^−1^ lime, the pH was about 8.9. A pH condition above 7.0 is said to be alkaline^25^. This is not the best pH condition to crop cowpea. Results^42^ have shown that just as low soil pH, severe alkaline soils will reduce the yield of cowpea. The reduced cowpea yield due to high pH could be due to the fact that: (1) certain essential nutrients may become less available to the cowpea plants. This is particularly true for micronutrients like iron, manganese, and zinc. Cowpeas, like other plants, require these micronutrients for proper growth and development. When these nutrients are less available, it can lead to nutrient deficiencies and reduced yield. (2) Soil pH influences the activity of soil microorganisms. Some microorganisms that contribute to nutrient cycling and availability may be less active or inhibited at higher pH levels. This can further exacerbate nutrient deficiencies for the cowpea plants. (3) High pH can affect the balance of various ions in the soil. For example, calcium and magnesium levels may increase at higher pH, potentially leading to an imbalance with other ions like potassium. This imbalance can negatively impact cowpea nutrient uptake and physiological processes. Goenaga et al.^43^ reported that lower cowpea yield could be as a result of lime-induced chlorosis brought about by high levels of calcium and magnesium carbonates in the soil. A very severe alkaline condition can also poise the crop to experience some diseases such as very severe leaf chlorosis. Seed germination might occur in a very alkaline condition but the growth of such plant afterward will be stunted without producing pods or death of the crop eventually.

## Conclusion

The results of these field experiments showed that the application of lime and biochar alone and in combination improved soil chemical characteristics (pH, N, P, K, Ca, Na, Mg, and CEC), growth (vine length per plant, number of leaves per plant, number of branches per plant), nodulation and yield parameters of cowpea (grain yield per plant and number of pods per plant) compared to the control. At higher levels of lime (10 t ha^−1^) with biochar, there were drastic yield reductions of cowpea, the reduction in the yield of cowpea can best be explained by the presence of unfavorable soil pH conditions. The optimum yield of the cowpea crop was achieved at 2.5 t ha^−1^ of lime with 10 t ha^−1^ of biochar. However, since the differences between the effects of 2.5 t ha^−1^ lime + 5 t ha^−1^ biochar, 2.5 t ha^−1^ lime + 7.5 t ha^−1^ biochar, and 2.5 t ha^−1^ lime + 10 t ha^−1^ biochar applications on cowpea yield were not significant, therefore to prevent waste of Biochar, 2.5 t ha^−1^ lime + 5 t ha^−1^ biochar is recommended for production of cowpea.

## Materials and methods

### Site description and experimental layout

We conducted two field experiments in the cropping seasons of 2020 and 2021 at the Teaching and Research Farm of Landmark University in Omu-Aran, Kwara State. Landmark University is situated within the coordinates Latitude 8° 7′ 26.21388″ and Longitude 5° 5′ 0.1788″, in an area characterized by undulating and rugged terrain with varying slopes and good overall drainage^27^. The main objective of these experiments was to assess how applying biochar and lime separately and together affects soil chemical properties, growth, nodulation, and yield of cowpea in tropical derived savanna Alfisol. The region experiences an annual rainfall of approximately 1300 mm and an average annual minimum and maximum temperature of 22 °C and 32 °C, respectively. There are two distinct rainy seasons: one from March to July and another from September to November. The peak of rainfall occurs in June and October. The experimental soil is categorized as Alfisol, specifically Oxic Haplustalf according to the USDA soil classification system^44^ and sandy loam in texture.

The treatments in both years (2020 and 2021) consisted of a factorial combination of five (5) levels (0, 2.5, 5.0, 7.5, and 10 t ha^−1^) of lime {calcium carbonate (CaCO_3_)} and five (5) levels (0, 2.5, 5.0, 7.5 and 10 t ha^−1^) of wood biochar. The 25 treatments were laid out with three blocks and three replications following factorial Randomized Complete Block Design. Each block comprised 25 plots and each plot measured 2 × 2 m. Spacing between blocks was 1 m apart while the spacing between plots was 1 m apart. Different sites were used for 2020 and 2021 experiments.

### Biochar incorporation, application of lime and crop establishment

The study utilized biochar derived from hardwood *(Prosopis africana*), a prevalent tree in the derived savanna. The hardwood *(Prosopis africana*) was collected break into small pieces to facilitate pyrolysis. The wood was packed in a metal drum (70 cm height and 46 cm diameter) that has small holes to allow gases to escape during pyrolysis. The lid was placed on the drum and was sealed tightly to minimize the entry of oxygen and was then ignited using natural gas. Gradually the temperature increased to around 500 °C. As the temperature rises, pyrolysis occurs inside the drum. Afterward, the resulting biochar underwent cooling, grinding, and sieving through a 2 mm sieve before being applied^3^.

Land preparation was done following traditional plowing and harrowing. Afterward, the experimental site was laid out to the required plot size of 2 × 2 m. Immediately after land preparation, predetermined amounts of biochar and lime (ranging from 0 to 10 t ha^−1^) were evenly spread on the plots, equivalent to 0 to 2.0 kg plot^−1^. The incorporation of these materials was accomplished using a hoe, reaching a depth of approximately 10 cm. This activity was carried out 2 weeks prior to sowing cowpea seeds.

The sowing of cowpea seeds (Variety Paiyur 1Cowpea) occurred on August 20th each year which falls within the growing season of cowpea in Kwara State, Nigeria. Two seeds were sown manually at the seed rate of 25 kg ha^−1^ to the depth of about 4 cm with an inter-row spacing of 20 cm and intra-row spacing of 75 cm. Subsequently, thinning was performed, allowing one plant per stand, ensuring a plant population of 27 plants per plot and 66,666 plants ha^−1^ for the erect variety. Two manual weedings were conducted throughout the experiment's duration. No irrigation water was applied. To control insect pests, cypermethrin was sprayed weekly at a rate of 30 mL per 10 L of water, commencing 2 weeks after sowing.

### Determination of soil and biochar properties

Each year, prior to the application treatments, soil samples were collected from a depth of 0–15 cm at ten random points within each experimental site. The collected soil samples were combined, air-dried, and passed through a 2 mm sieve for subsequent analysis of soil physical and chemical properties. Particle size analysis was conducted using the hydrometer method as described by Gee and Or^45^. Soil organic carbon (OC) was quantified following the Walkley and Black procedure, utilizing the dichromate wet oxidation method outlined by Nelson and Sommers^46^. Total nitrogen (N) was determined through the micro-Kjeldahl digestion method as detailed by Bremner^47^. Available phosphorus (P) was assessed using Bray-1 extraction followed by molybdenum blue colorimetry, following the protocol of Frank et al.^48^. Exchangeable potassium (K), sodium (Na), calcium (Ca), and magnesium (Mg) were extracted using 1 M ammonium acetate, as outlined by Hendershot et al.^49^. Subsequently, K concentration was determined using a flame photometer, while Na, Ca, and Mg concentrations were analyzed using an Atomic Absorption Spectrophotometer. Soil pH was measured using a soil–water mixture at a 1:2 ratio, and the measurement was taken using a digital electronic pH meter^50^. Exchange acidity was determined as described by Udo et al.^51^ by weighing 5 g of dried soil into a sample bottle, adding 50 mL of 1 M KCl, shaking the mixture for an hour, and then filtering the suspension to obtain a filtrate. To the extract in a 100 mL conical flask, four drops of phenolphthalein indicator were added. The mixture was titrated with 0.01 M NaOH until the color shifted from colorless to pink, marking the endpoint.

$${\text{Exchangeable}}\;{\text{acidity}} = \frac{{({\text{T}}{-}{\text{B}}) \times {\text{C}}_{{{\text{NaOH}}}} \times {\text{V}}_{1} \times 100}}{{{\text{Weight}}\;{\text{of}}\;{\text{soil}} \times {\text{V}}_{2} }}$$

where T stands for sample titre value in milliliters (mL), B for blank titre value in mL, W for the weight of soil in grams (g), V_1_ for the volume of extracting solution in mL, V_2_ for the volume of soil extract in mL, and C_NaOH_ for the standardized concentration of NaOH (0.002 M NaOH). The summation approach was used to calculate the ECEC in this study, which is the total of the exchangeable bases and exchangeable acidity^52^.

We conducted excavations for three plants, keeping their rhizosphere soils intact. This excavation occurred when the cowpea plants had reached 50% flowering on a plot-wise basis, approximately 65 days after sowing. Nodules present in the roots of the excavated plants were counted using a magnifying glass. Post-experiment, we collected soil samples on a per-plot basis. Subsequently, these soil samples were air-dried, sieved using a 2 mm sieve, and underwent analysis for soil chemical properties using the aforementioned procedures.

For this experiment, we subjected the biochar to analysis to determine its nutrient composition. The biochar was air-dried and sieved using a 2 mm sieve. Following this preparation, we conducted analyses for organic carbon (OC), nitrogen (N), phosphorus (P), potassium (K), calcium (Ca), and magnesium (Mg) in accordance with AOAC^53^ standards. Additionally, we determined the pH of the biochar using a 1:20 suspension of biochar to water, following the method described by Rajkovich et al.^52^.

### Determination of growth and yield parameters

#### Growth metrics

Data regarding the growth of cowpea plants was obtained during the mid-flowering stage, precisely 65 days after planting. The assessment involved counting the fully expanded leaves to determine leaf count, using a measuring tape to measure shoot length, and counting the branches on each plant to ascertain the number of branches.

#### Yield metrics

The cowpea (Variety Paiyur 1Cowpea) used was a determinate type. When the cowpea pods reached maturity (about 100 days after sowing), they were harvested and the number of harvested pods was tallied and documented for each plot and treatment. To determine grain yield, matured cowpea pods were harvested, shelled and weighed using a precise weighing balance, and the weight was recorded for each plot.

### Statistical analysis

The collected data underwent a statistical analysis of variance (ANOVA) utilizing the Statistical Package for Social Sciencesy^54^. Treatment means were compared using the Duncan Multiple Range Test (DMRT) at a significance level of 0.05.

### Ethical approval

I confirm that all the research meets ethical guidelines and adheres to the legal requirements of the study country.

### Compliance with international, national and/or institutional guidelines

Experimental research (either cultivated or wild), comply with relevant institutional, national, and international guidelines and legislation. Experimental studies were carried out in accordance with relevant institutional, national or international guidelines or regulation.

## Data Availability

All datasets generated and/or analysed during the current study are included in this article.

## References

[1] Robarge, W. P. Acidity. In: *Encyclopedia of Soil Science, Encyclopedia of Earth Sciences Series* (ed Ward Chesworth) 860pp (2008).

[2] Kochian V, Hoekenga A, Pineros A (2004). How do crop plants tolerate acid soils? Mechanisms of aluminum tolerance and phosphorous efficiency. Annu. Rev. Plant Biol..

[3] Adekiya AO (2022). Improving tropical soil productivity and cowpea (*Vigna unguiculata* (L.) Walp) performance using biochar and phosphorus fertilizer. Commun. Soil Sci. Plant Anal..

[4] Menzies NW, Rengel Z (2003). Toxic elements in acid soils: Chemistry and measurement. Handbook of Soil Acidity.

[5] Sylvia, D. M., Fuhrmann, J. J., Hartel, P. G. & Zuberer, D. A. *Principles and Applications of Soil Microbiology (No. QR111 S674 2005)* (Pearson Prentice Hall, Upper Saddle River, NJ) (2005).

[6] Ano AO (2006). Effect of vegetable cowpea population on component crop yields and productivity of yam based system. Niger. J. Agric..

[7] Gatiboni, L. & Hardy, D. *Soil Acidity and Liming: Basic Information for Farmers and Gardeners*. AG-439-51, North Carolina Cooperative Extension (2003).

[8] Anetor MO, Akinrinde EA (2007). Lime effectiveness of some fertilizers in a tropical acid alfisol. J. Cent. Eur. Agric..

[9] Lehmann J, Rondon M, Uphoff N, Ball AS, Fernandes E, Herren H, Husson O, Laing M, Palm C, Pretty J, Sanchez P, Sanginga N, Thies J (2006). Bio-char soil management on highly weathered soils in the humid tropics. Biological Approaches to Sustainable Soil Systems.

[10] Chintala R, Mollinedo J, Schumacher TE, Malo DD, Julson JL (2014). Effect of biochar on chemical properties of acidic soil. Arch. Agron. Soil Sci..

[11] Juriga M, Šimanský V (2019). Effects of biochar and its reapplication on soil pH and sorption properties of silt loam Haplic Luvisol. Acta Hortic. Regiotect..

[12] Shetty R, Prakash NB (2020). Effect of different biochars on acid soil and growth parameters of rice plants under aluminium toxicity. Sci. Rep..

[13] Lin Q, Zhang L, Riaz M, Zhang M, Xia H, Lv B, Jiang C (2018). Assessing the potential of biochar and aged biochar to alleviate aluminum toxicity in an acid soil for achieving cabbage productivity. Ecotoxicol. Environ. Saf..

[14] Onwerenmadu EI, Opara CC, Duruigbo CI, Ihejirika CO (2003). Effect of different rate of dolomite limestone on the grain yield of white brown. Afr. J. Environ. Stud..

[15] Muleba, N., Dabire, C., Suh, J. B., Drabo, I. & Ouedraogo, J. T. Technologies for cowpea production based on genetic and environmental manipulations in the semi-arid tropics. Pages 195–206 in Technology options for sustainable agriculture in sub-Saharan Africa. In: *Publication of the semi-arid food grain research and development agency (SAFGRAD) of the scientific* (eds. Bezuneh, T., Emechebe, A. M., Sedgo, J. & Ouedraogo, M.) 195–206 (Technical and Research Commission of OAU, Ouagadougou, Burkina Faso, 1997).

[16] Osipitan OA, Fields JS, Lo S, Cuvaca I (2021). Production systems and prospects of cowpea (*Vigna unguiculata* (L.) Walp.) in the United States. Agronomy.

[17] Oyinlola EY, Chude VO (2010). Status of available micronutrients of the basement complex rock—Derived Alfisols in northern Nigeria savanna. Trop. Subtrop. Agroecosyst..

[18] Sani, M., Alhassan, I., Aduloju, M. O., Hegarty, P. J. & Musa, A. A. Spatial variability of soil pH and primary nutrients of Alfisols in parts of the Savannas of Yobe State, Nigeria. In *Proceedings of 37*^*th*^* Annual Conference of the Horticultural Society of Nigeria “OWO MADE 2019, 18*^*th*^*–22 November, 2019*, pp. 494–500 (2019)

[19] IITA 1998 International Institute of Tropical Agriculture (IITA). Grain legumes Improvement Programme. Annual Report for 1986, Ibadan, Nigeria. p. 3–5.

[20] Yeng SB, Agyarko K, Dapaah HK, Adomako WJ, Asare E (2012). Growth and yield of sweet potato (*Ipomoea batatas* L.) as influenced by integrated application of chicken manure and inorganic fertilizer. Afr. J. Agric. Res..

[21] Nedunchezhiyan M, Reddy D (2002). Nitrogen management in sweet potato (*Ipomoea batatas*) under rainfed conditions. Indian J. Agron..

[22] Martí HR, Mills HA (2002). Nitrogen and potassium nutrition affect yield, dry weight partitioning, and nutrient use efficiency of sweet potato. Commun. Soil Sci. Plant Anal..

[23] Niu Z, Ma J, Fang X, Xue Z, Ye Z (2022). Effects of application of rice husk biochar and limestone on cadmium accumulation in wheat under glasshouse and field conditions. Sci. Rep..

[24] Adekiya AO, Ayorinde BB, Alori ET, Aremu C, Ejue WS (2023). Effects of lime on soil chemical characteristics and performance of cowpea [*Vigna unguiculata* (L.) Walp.] on Oxic Haplustalf of a derived savanna ecology of Nigeria. Res. Crop.

[25] FFD (2011). (Federal Fertilizer Department). Fertilizer Use and Management Practices for Crop Production in Nigeria.

[26] Akinrinde, E. A. & Obigbesan, G. O. Evaluation of the fertility status of selected soils for crop production in five ecological zones of Nigeria. In *Proceedings of the 26th Annual Conference of Soil Science Society of Nigeria* (ed Babalola, O.), 279–88 (30–3 October November, Ibadan, Nigeria, 2000).

[27] Adegbite KA, Adekiya AO, Adebiyi OTV, Alori ET, Ejue WS, Olayanju A, Aremu C (2020). Baseline fertility status of a gravelly Alfisol in a derived savannah agro-ecological zone of Nigeria. Open Agric..

[28] Ameyu T (2019). A review on the potential effect of lime on soil properties and crop productivity improvements. J. Environ. Earth Sci..

[29] Kisinyo PO, Othieno CO, Okalebo JR, Kipsat MJ, Serem AK, Obiero DO (2005). Effects of lime and phosphorus application on early growth of *Leucaena* in acid soils. Afr. Crop Sci. Conf. Proc..

[30] Glaser B, Lehmann J, Zech W (2002). Ameliorating physical and chemical properties of highly weathered soils in the tropics with charcoal—A review. Biol. Fertil. Soils.

[31] Nigussie A, Kissi E, Misganaw M, Ambaw G (2012). Effect of aiochar application on soil properties and nutrient uptake of lettuces (*Lactuca sativa*) grown in chromium polluted soils. Am.-Eurasian J. Agric. Environ. Sci..

[32] Abdeen SA (2020). Biochar, bentonite and potassium humate effects on saline soil properties and nitrogen loss. Annu. Res. Rev. Biol..

[33] Miller, J. S., Rhaodes, A. & Puno, H. K. Plant nutrient in biochar. Adv. Agro: 125 (2012).

[34] Jia X, Yuan W, Ju X (2015). Short report: Effects of biochar addition on manure composting and associated N_2_O emissions. J. Sustain. Bioenergy Syst..

[35] Jones DL, Rousk J, Edwards-Jones G, DeLuca TH, Murphy DV (2012). Biochar-mediated changes in soil quality and plant growth in a three year field trial. Soil Biol. Biochem..

[36] Wang L, Butterly CR, Wang Y, Herath HMSK, Xi YG, Xiao XJ (2014). Effect of crop residue biochar on soil acidity amelioration in strongly acidic tea garden soils. Soil Use Manag..

[37] Agusalim M, Wani HU, Syechfani MS (2010). Rice husk biochar for rice based cropping system in acid soil: The characteristics of rice husk biochar and its influence on the properties of acid sulfate soils and rice growth in West Kalimantan, Indonesia. J. Agric. Sci..

[38] Adekiya AO, Agbede TM, Olayanju A, Ejue WS, Adekanye TA, Adenusi TT, Ayeni JF (2020). Effect of biochar on soil properties, soil loss, and cocoyam yield on a tropical sandy loam Alfisol. Sci. World J..

[39] Haruna IM (2011). Dry matter partitioning and grain yield potential in Sesame (*Sesamum indicum* L.) as influence by Poultry manure, nitrogen and phosphorus at Samaru, Nigeria. Elixir Agric..

[40] Adekiya AO, Adebiyi OV, Ibaba AL, Aremu C, Ajibade RO (2022). Effects of wood biochar and potassium fertilizer on soil properties, growth and yield of sweet potato (*Ipomea batata*). Heliyon.

[41] Appunu C, Dhar B (2006). Symbiotic effectiveness of acid-tolerant Bradyrhizobium strains with soybean in low pH soil. Afr. J. Biotechnol..

[42] Soares BL, Ferreira PAA, de Oliveira-Longatti SM, Marra LM, Rufini M, de Andrade MJB, de SouzaMoreira FM (2014). Cowpea symbiotic efficiency, pH and aluminum tolerance in nitrogen-fixing bacteria. Sci. Agricola.

[43] Goenaga R, Gillaspie AG, Quiles A (2010). Field screening of cowpea genotypes for alkaline soil tolerance. Hortscience.

[44] USDA. *Soil Taxonomy Soil Survey Staff, Agriculture Handbook, No 436*. 2nd ed (United States Department of Agriculture, Natural Resources Conservation Service, Washington, DC) 869 (1999).

[45] Gee, G. W. & Or , D. Particle-size analysis. In *Methods of Soil Analysis, Part 4* (eds Dane, J. H. & Topp, G. C.) 255–93 (Physical Methods, Madison). Soil Science Society of America Book Series No. 5 (2002).

[46] Nelson, D. W. & Sommers, L. E. Total carbon, organic Carbon and organic matter. In *Methods of Soil Analysis Part 3—Chemical Methods* (eds Sparks, D. L., Page, A. L., Helmke, P. A. & Loeppert, R. H.) 961–1010 (Soil Science Society of America, Americal Society of Agronomy, Madison, 1996).

[47] Bremner, J. M. Nitrogen-total. In *Methods of Soil Analysis. Part 3. Chemical Methods* (ed Sparks, D. L.) 2nd edn, SSSA Book Series No. 5., 1085–121 (ASA and SSSA, Madison, 1996).

[48] Frank, K., D. Beegle, and J. Denning, 1998. Phosphorus. In Recommended Chemical Soil Test Procedures for the North Central Region, North Central Regional Research, ed. Brown J. R., Revised, 21–26. Columbia: Missouri Agric. Exp. Station. Publication No. 221. (1998).

[49] Hendershot, W. H., Lalande, H. & Duquette, M. Ion exchange and exchangeable cations. Soil sampling and methods of analysis. In *Canadian Society of Soil Science,* 2nd edn. Chapter 18 (eds Carter, M. R. & Gregorich, E. G.) 197–206 (International Institute of Tropical Agriculture, Ibadan, Nigeria, CRC Press, Boca Raton (Florida), 2007).

[50] Ibitoye AA (2006). Laboratory Manual on Basic Soil Analysis.

[51] Udo EJ, Ibia TO, Ogunwale JA, Ano AA, Esu IE (2009). Manual of Soil, Plant and Water Analyses.

[52] Rajkovich S, Enders A, Hanley K, Hyland C, Zimmerman AR, Lehmann J (2012). Corn growth and nitrogen nutrition after additions of biochars with varying properties to a temperate soil. Biol. Fertile Soils.

[53] AOAC 2012 Official methods of analysis of the association of official analytical chemists. In AOAC International (ed Latimer G. W.), 19th edn, 2–15 (AOAC International, Gaithersburg, MD).

[54] SPSS Inc. Released 2009. PASW Statistics for Windows, Version 18.0 (SPSS Inc., Chicago, 2009).

